# T108. ANALYTICAL AND PREDICTIVE VALIDITY OF HALLUCINATIONS IN FIRST PSYCHOTIC EPISODES

**DOI:** 10.1093/schbul/sby016.384

**Published:** 2018-04-01

**Authors:** Maria Hernandez-Garcia, Carlos Gonzalez, Pablo Soldevilla, Olga Sparano, Miguel Hernandez, Carlos Cañete, Julio Sanjuan

**Affiliations:** 1 University of Valencia; 2 Clinic Hospital, Valencia INCLIVA; 3 Rivera Hospital; 4 Clinic Hospital, Valencia INCLIVA, CIBERSAM

## Abstract

**Background:**

Some studies of first psychotic episodes have suggested the association between childhood trauma, such as sexual abuse, and the risk of hallucinations.1 Furthermore, other studies indicated that environment can alter the phenomenological presentation of first psychotic episodes.2

However, there are no studies about the association between hallucinations in first psychotic episodes and the prognosis of the disease. This is the main objective of this study. We also compared the phenomenological differences between hallucinations in first episode psychosis and persistent hallucinations in patients with chronic psychosis.

**Methods:**

Naturalistic, longitudinal follow-up study in a sample of 173 patients of first psychotic episode attending public mental health service in Area 5 of Valencia region (Spain) in a period between 2010–2017.

We compared first-episode patients with hallucinations (N=38) with two samples: A) First-episode patients without hallucinations (N=137). B) Chronic patients with persistent hallucinations (N=45) from a previous study.3

In the first comparison we used the following variables: sociodemographic data, risk factors (such as cannabis consume and immigration), psychiatric pathology (CIE-10), psicopathology (including clinical scales GAF, CGI and PANSS), number of emercengy visits and number of hospitalization after the first psychotic episode.

In the second one, we use the PSYRATS scale to compare both groups.

**Results:**

In the first comparison, First Episode Psychotic patients with and without hallucinations,we only found significant differences in the number of hospital income, with more hospitalizations in the non hallucinating group (P= 0.001).

In the second comparison, First Episode Hallucinations versus Chronic Persistent Hallucinations, significant differences were only found in the duration of the hallucinations, which was much higher in chronic persistent hallucinations group (P= 0.001)

**Discussion:**

Consequently, it seems that first psychotic episode patients without hallucinations have more hospitalizations than first-episode patients with hallucinations. Moreover, we can conclude that the duration of voices is higher in chronic patients with persistent hallucinations than in first psychotic episode hallucinations.

Both results have practical implications in the prognostic importance of hallucinations in first psychotic episodes.

**References:**

1. Misiak, B. et al “Childhood traumatic events and types of auditory verbal hallucinations in first-episode schizophrenia patients”, Comprehensive Psychiatry 66 (2016) 17–22.

2. Oher, F.J. et al “The effect of the environment on symptom dimensions in the first episode of psychosis: a multilevel study”, Psychological Medicine 44 (2014) 2419–2430.

3. González, J.C. et al J., “Persistent Auditory Hallucinations”, Psychopathology 39 (2006) 120–125.

